# Evaluation of university and training standards in clinical perfusion, an European-wide survey

**DOI:** 10.1093/icvts/ivae134

**Published:** 2024-08-08

**Authors:** André Giesbrecht, Christian Klüß, Gerdy Debeuckelaere, Maria Angeles Bruño, Folker Wenzel, Matthias Kohl, Filip De Somer, Adrian Bauer

**Affiliations:** European Board of Cardiovascular Perfusion, Brussels, Belgium; Faculty Medical and Life Sciences, Furtwangen, University of Applied Sciences, Campus Villingen-Schwenningen, Germany; Department of Thoracic and Cardiovascular Surgery, Clinic for Thoracic and Cardiovascular Surgery, Herz- und Diabeteszentrum NRW, Ruhr-Universität Bochum, Bad Oeynhausen, Germany; European Board of Cardiovascular Perfusion, Brussels, Belgium; Department of Thoracic and Cardiovascular Surgery, Clinic for Thoracic and Cardiovascular Surgery, Herz- und Diabeteszentrum NRW, Ruhr-Universität Bochum, Bad Oeynhausen, Germany; European Board of Cardiovascular Perfusion, Brussels, Belgium; Department for Perfusion, University Hospital Antwerp, Edegem, Belgium; Department for Perfusion, Consorcio Hospital General Universitario Valencia, Valencia, Spain; Faculty Medical and Life Sciences, Furtwangen, University of Applied Sciences, Campus Villingen-Schwenningen, Germany; Faculty Medical and Life Sciences, Furtwangen, University of Applied Sciences, Campus Villingen-Schwenningen, Germany; European Board of Cardiovascular Perfusion, Brussels, Belgium; Department for Perfusion, Heart Center, University Hospital Ghent, Ghent, Belgium; European Board of Cardiovascular Perfusion, Brussels, Belgium; Department for Perfusion, Evangelic Heartcenter Coswig, Coswig, Germany

**Keywords:** Clinical perfusionist, Education, Harmonized training standards, Cardiovascular perfusion, European Board of Cardiovascular Perfusion, European Credit Transfer System

## Abstract

**OBJECTIVES:**

Adequate theoretical and practical training of prospective clinical perfusionists is essential for maintaining clinical standards and ensuring patient safety during cardiac surgery procedures. Perfusion schools play a crucial role in establishing and maintaining higher education and training standards in clinical perfusion. The aim of this study is to obtain a comprehensive overview of European training standards in clinical perfusion in 2023.

**METHODS:**

For this study, 53 perfusion schools in Europe were found and contacted, of which 30 (56.6%) responded, giving a sample size of *n* = 30, which were then included in the data analysis. The quantitative data of the survey are analysed using descriptive methods.

**RESULTS:**

The university and training standards in clinical perfusion in Europe vary in many respects. Starting with the entry criterion for studies (most frequently a required bachelor’s degree 36.7% or 2nd most common an university entrance qualification 30%), the duration [from <12 months (13.3%) up to 36 months (13.3%)] and regarding the content of the teaching in clinical perfusion [<30 European Credit Transfer System (ECTS) (33.3%) and more than 180 ECTS (6.7%)]. The mean value for teaching in clinical perfusion content is 62.63 ECTS credits.

**CONCLUSIONS:**

The obtained results show important differences between countries and schools. As such, they form a valuable database for future discussions establishing a common European curriculum and training standards for perfusionists. For the generalizability of the results, further evaluations and larger samples are needed.

## Graphical Abstract

**Figure:**
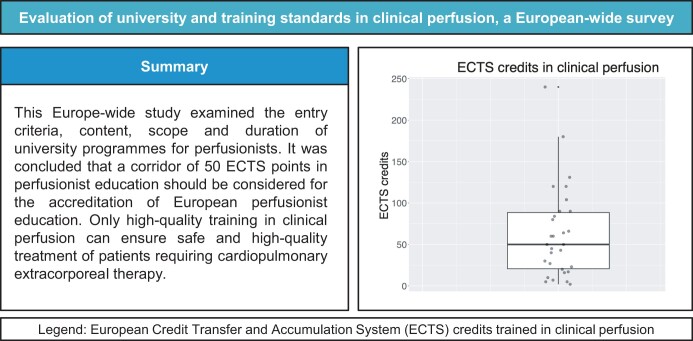

## INTRODUCTION

The profession of clinical perfusion can be attributed to the achievements of the American surgeon J.H. Gibbon Jr. who was historically the 1st to successfully use the heart–lung machine during cardiac surgery [1]. Since then, the profession has developed from a technical assistant to an indispensable team member of the ‘heart team’. According to Bauer *et al.*, a clinical perfusionist is able to plan, prepare and perform extracorporeal circulations in a patient-oriented manner by combining medical and technical knowledge. Clinical perfusion is to a large part the result of multidisciplinary teamwork, which is carried out on a physician’s order. Nowadays, the professional activity of a perfusionist is no longer limited to conventional cardiopulmonary bypass but comprises many other techniques such as minimally invasive extracorporeal circulation [2], mechanical cardiovascular support, organ transplantation, isolated organ perfusion and participation in interventional cardiology procedures. Apart from the previous clinical activity, perfusionists are involved in research and development of new equipment and procedures [3]. Summarizing Merkle describes clinical perfusion as a combination of technical expertise and medical knowledge [4].

Despite the high level of expertise of perfusionists, acknowledged by many cardiac surgery units throughout Europe, there is a lack of adequate harmonized training standards.

The European Board of Cardiovascular Perfusion (EBCP), founded in 1991, has the main goal of creating, establishing, monitoring and maintaining European-wide training standards in clinical perfusion [5]. The last evaluation of training standards in clinical perfusion (2006) shows that training programmes differ in various aspects [6]. In order to get an up-to-date overview of the education and training standards in clinical perfusion in Europe, the EBCP requested the working group for this survey. The study was part of a master thesis at the University of Furtwangen.

Merkle's European-wide survey (2006) concludes that education and training in Europe is nor comparable nor harmonized [6]. A variety of quite different curricula and training models continue to exist, ranging from semi-skilled workers to vocational training programmes and academic programmes offering Bachelor’s and Master’s degrees as well as other academic degrees [6].

In order to establish and monitor European-wide education standards in clinical perfusion, the EBCP needs the cooperation of various partners involved in the treatment and care of cardiovascular patients and clinical perfusion. The European Association for Cardio Thoracic Surgery (EACTS)—European Association of Cardiothoracic Anaesthesiology and Intensive Care (EACTAIC)—EBCP guidelines published in 2019 explicitly call for adequate training combined with appropriate knowledge, skills and expertise for perfusionists. Although the guidelines refer specific to the EBCP, as the reference for setting standards for theoretical and practical training, only a limited number of countries have implemented the recommended standards of the EBCP. In order to reach competences in clinical perfusion, perfusionists must achieve special knowledge, skills and techniques. In order to get this level, it is generally agreed that both formal theoretical and practical training is required [7]. Another important contribution is made by the associations of national professional societies that are indirectly or directly related to the perfusion profession (societies of perfusionists, cardiothoracic surgeons, cardiologists, anaesthetists and intensive care physicians) in the form of expert statements, consensus papers and guideline recommendations. For example, all relevant German professional societies recommend aiming for a uniform academic degree of level 6 (Bachelor) for the training of perfusionists according to the German (European) qualifications framework [3]. For Gummert *et al.*, holders of the European Certificate in Cardiovascular Perfusion (ECCP), are among the basic requirements of a specialist cardiac surgery department in Germany [8].

Despite the EBCP’s cooperation with various national and international partners, harmonization of training in perfusion can only be realized when an important partner is more involved, namely the perfusion schools.

A perfusion school is responsible for teaching theoretical perfusion-related content in addition to the practical training phase in a hospital. Due to major differences across Europe in terms of entry requirements, training model, duration and comprehensiveness of training, the lowest common denominator has so far been taken as standard by the EBCP (since 1991), defined as:

1. Teaching of perfusion-related subjects in the curriculum
2. Documentation of at least 100 clinical perfusions (supervized perfusion with logbook)
3. Central theoretical final EBCP examination [9]

Since the quality standards required by the EBCP, today, do not provide any details on the required curriculum and as no potential curricula have been published, an European-wide survey is required. The results of this survey will provide detailed information on the curricula of existing perfusion schools. The data will provide a basis for discussion at the European level and serve as a basis for further development of training and education in clinical perfusion.

## METHODS

In collaboration with Furtwangen University, an online survey has been conducted in Europe between 22 November 2022 and 15 January 2023. Microsoft Forms have been used for this survey, which is included in the Microsoft Office 365 package and is kindly provided by the ‘German Society for Cardiovascular Engineering’ (DGfK). The survey consists of 18 questions, a 1st part queries about essential aspects of training in clinical perfusion, such as entry requirements, status of accreditation according to EBCP, final examination and final qualification, a 2nd part allows input for ideas and improvements in the curriculum. The study population consisted of all perfusion schools in geographical Europe (the EBCP affiliated country South Africa is included). Only perfusion schools primarily targeting the conduction of cardiopulmonary bypass were included. Extra Corporeal Membrane Oxygenation (ECMO) courses were not included. Lack of accreditation of a school by EBCP was not an exclusion criterion.

Since not all schools *n* = 13 (43.3%) were able to express the amount of teaching content in European Credit Transfer System (ECTS) credits, a data transformation was carried out. For perfusion schools where the ECTS credits were not provided, hours were multiplied by the factor 1/30 (1 ECTS credit ≙ 25–30 h study workload).

Contact addresses of perfusion schools were provided by the national delegate of each country. Each perfusion school was contacted at least 2 or 3 times. In summary, the population to be investigated was *n* = 53 perfusion schools, of which a sample with a sample size of *n* = 30 is included in the data collection. Afterwards, the data are characterized with the help of descriptive statistics using ‘R’ (R Core Team) [10, 11].

## RESULTS

Out of the contacted perfusion schools in Europe (*n* = 53), only 30 (56.6%) participated in the survey.

### Study type and accreditation

The most common forms of education in perfusion in Europe are either undergraduate training [12 (40.0%)] or part-time apprenticeships [11 (36.8%)] after already having completed initial training. Postgraduate full-time courses are less common [7 (23.3%)]. The majority of perfusion schools are accredited by the EBCP as a suitable training site [18 (60.0%)]. More than half of the remaining non-accredited schools [12 (40.0%)], have never been accredited by the EBCP [7 (58.25%)].

### Entry-level requirements

Most perfusion schools require a bachelor’s degree to start the study programme [11 (36.7%)]. The 2nd most common entry-level requirement is proof of university entrance qualification (Abitur, A-Level, Baccalaureate) [9 (30.0%)].

Of the 30 participating schools, only *n* = 9 (30%) comment on a required work experience in a previously completed health profession. Two years [4 (44.4%)] or 1 year of work experience is required, if at all.

### Final examination

**Figure 1:**
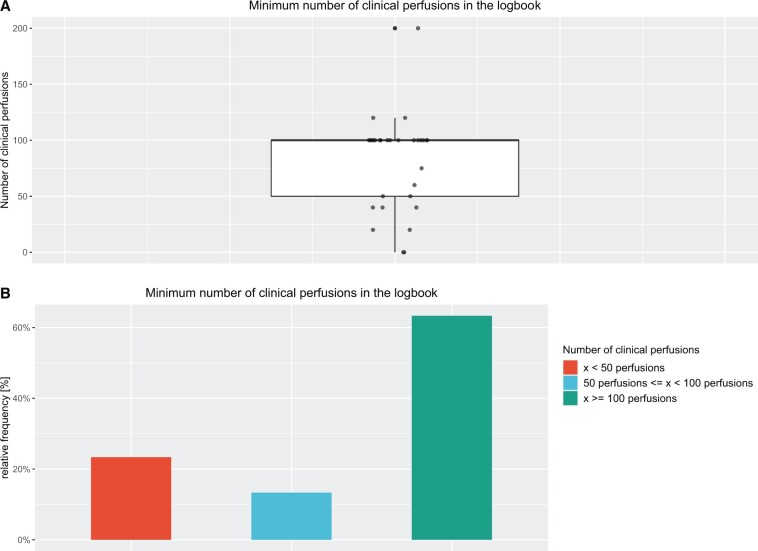
(**A**) Box plot for plotting clinical perfusions required in the logbook; (**B**) plot of relative frequencies of clinical perfusions in the logbook.

Most perfusion schools [19 (63.3%)] require a logbook with at least a minimum of 100 witnessed clinical perfusions before being eligible for qualification. However, almost a quarter of schools require *x* < 50 clinical perfusions in the logbook [7 (23.3%)]. All data including variations are illustrated in Table 1 and in Fig. 1. The median is *x* = 100 clinical perfusions; 25% of the values are in the 1st quartile between 50 and 100 clinical perfusions. Since the median is larger than the mean, there is a left-skewed distribution. The outliers of 0 in contrast to 200 required clinical perfusions in the logbook are remarkable. Both the interquartile range of 50, the range of 200 and the standard deviation of 41.93 witnessed clinical perfusions illustrate the wide dispersion of the values.

**Table 1:** Position and dispersion parameters of some perfusion-related variables

Position and scattering parameters	Number witnessed clinical perfusions (/)	Duration of study programme hours (h)	ECTS credits study programme	ECTS credits taught in clinical perfusion	Hours taught in clinical perfusion (h)
	*n* = 30	*n* = 30	*n* = 24	*n* = 30	*n* = 30
Minimum	0	200	20	2	60
Maximum	200	6300	240	240	6000
Median	100	2250	120	50	1275
First quartile	50	1262.5	80	20.75	600
Third quartile	100	4275	180	88.5	2168
IQR	50	3012.5	100	67.75	1567.5
Skew	0.04	0.59	0.25	1.32	1.24
Mean	81.17	2824.93	131.72	62.63	1612.83
Range	200	6100	220	238	5940
Standard deviation	41.93	1989.07	69.46	55.14	1457.55

ECTS: European Credit Transfer System.

The accredited perfusion schools most frequently award an ECCP certificate without an academic degree [9 (52.9%)], while the non-accredited schools most frequently award a Bachelor’s degree or a state-recognized certificate [4 each (30.8%)]. According to the classification of European Qualifications Framework (EQF for lifelong learning), level 6 is awarded most frequently for accredited and non-accredited schools combined [14 (46.7%)]. It is distinctive that more than a quarter of degrees are master’s degrees (EQF level 7) [9 (30.0%)].

### Duration of study programmes

The duration of the surveyed study curricula in Europe varies significantly. Going from 6-month, full-year to 3 and 4-year curriculum, most of the study curricula had a total duration of more than 36 months [11 (36.7%)], followed by curricula with a study duration of 24 months or more [9 (30.0%)]. There are also programmes that last <12 months [4 (13.3%)].

The median is 2250 h, which would correspond to a number of 75 ECTS credits with a ratio of 30 h/ECTS credit. Since the median is smaller than the mean, there is a right-skewed distribution of the values. The outliers of very short programmes with a 200-h training duration are noticeable. Both the interquartile range of 3012.5 h and the range of 6100 h and the standard deviation of 1989.07 h illustrate the very large dispersion of the values.

### ECTS credit points study programme

Most European perfusion schools (*n* = 24; 80%) issue ECTS credits. The median is 120 ECTS credits. Since the median is somewhat smaller than the mean, there is a slightly right-skewed distribution of the values. Both the interquartile range of 100 ECTS credits and the range of 220 ECTS credits and the standard deviation of 69.46 ECTS credits illustrate the wide dispersion of the values.

### ECTS credits taught in clinical perfusion

**Figure 2:**
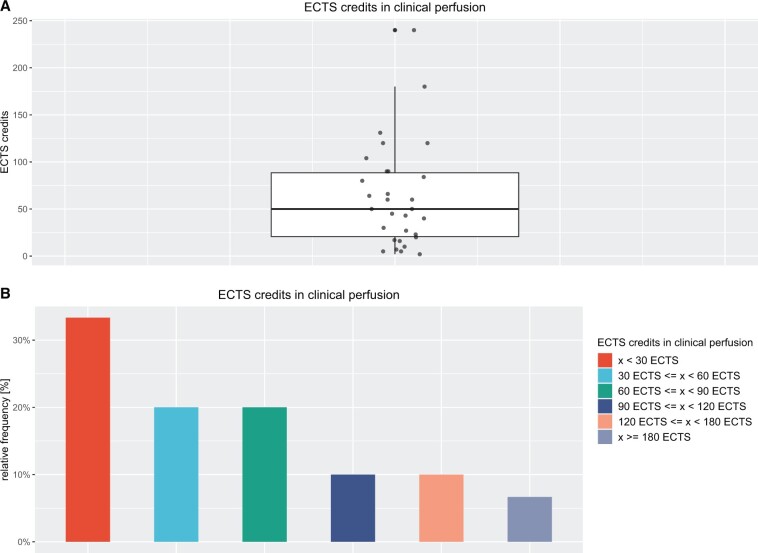
(**A**) Box plot for plotting ECTS credits in clinical perfusion; (**B**) plot of relative frequencies of ECTS credits in clinical perfusion. ECTS: European Credit Transfer System.

Most of the *n* = 30 perfusion schools show an amount of education in clinical perfusion of <30 ECTS credits [10 (33.3%)]. Table 1 and Fig. 2 illustrate the position and dispersion parameters. The median for *n* = 30 is 50 ECTS credits. Since the median is smaller than the mean, there is a right-skewed distribution of the values. The interquartile range of 67.75 ECTS credits and the range of 238 ECTS credits and the standard deviation of 55.14 ECTS credits illustrate the wide dispersion of the values. The outliers upwards and downwards with values of 240 ECTS credits versus 2 ECTS credits are noteworthy. Similar results are applicable to the number of hours taught in clinical perfusion (Table 1).

### Ideal curriculum

 

### Bivariate analysis

 

## DISCUSSION

There are essentially 2 different approaches to education and training in clinical perfusion in Europe: approach A (undergraduate) and B (postgraduate).

Approach A [12 (40.0%)] integrates clinical perfusion as a specialization in an engineering or nursing degree (undergraduate education). Approach B [11 (36.8%)] builds on an existing initial training in a health profession and has the character of a postgraduate part-time or, less frequently, full-time continuing education [7 (23.3%)]. Surprisingly, half of the perfusion schools accredited by the EBCP seem to favour teaching approach B in contrast to the non-accredited perfusion schools, the majority of which [7 (58.3%)] offer undergraduate academic training in clinical perfusion.

The results on entry-level requirements support Merkle’s statement that the majority of European training programmes in clinical perfusion regulate the entry of training through entry-level requirements [6]. While teaching approach A only requires a general university entrance qualification for initial academic training [9 (30.0%)], in many European countries, a bachelor’s degree is required as an entry requirement for postgraduate perfusion training [11 (36.7%)]. Thus, this approach already achieves the EQF level 6 for clinical perfusionists targeted by Bauer *et al.* via its entry requirements [3].

The differences between teaching approaches A and B are also evident in the conduct of final examinations. The high frequency of theoretical university final examinations is not only to be found in undergraduate study programmes. Postgraduate continuing education programmes also conclude as academic continuing education with an examination at the university, but without the award of an academic degree (Bachelor/Master).

More than half of the schools agree that the logbook should list a minimum of 100 clinical perfusions (Fig. 1). When looking for reasons why there are perfusion schools that only require a minimum of <50 perfusions in the logbook, bivariate analysis helps further. The Cramer’s V contingency coefficient (0.7066975) indicates a strong association between the status of EBCP accreditation and the minimum number of clinical perfusions in the logbook (Fig. 4B). This again confirms the assumption that in the non-accredited and often undergraduate training programmes, less emphasis is placed on the practical part of the training.

The findings on the total duration and comprehensiveness of training programmes are inconsistent. Wahba *et al.* have already noted in the Guidelines published in 2019 that the duration of training in Europe varies between 1 and 4 years [7]. Colligan and Patel note that so called ‘certificate programs’ generally have a shorter duration [12]. In general, the duration of teaching and training in clinical perfusion is relevant for the quality of the training programme if the minimum requirements of the curriculum are fulfilled. Therefore according to Merkle, a 2-year training programme is not appropriate and should be made to meet international standards [13]. Table 1 reveals many inconsistencies because the information on the total duration of the training in hours does not coincide with the amount of ECTS credits. With such a large spread of values, neither the mean nor the median can provide a meaningful benchmark.

The question about the required amount for teaching content is one of the core questions of this quantitative data collection. The authors of the study expect a better understanding of the teaching time in clinical perfusion expressed in ECTS credits. In view of the high dispersion of the values, neither the median value of 50 nor the mean value of 62.63 ECTS credits are useful as a target value for a definite recommendation of the scope of education and training in clinical perfusion. This was not the aim of the study, rather the authors wanted to get a clearer picture of the perfusion training situation in Europe. This was successful insofar as it shows the need to describe the necessary competences for perfusionists more concisely and to demand the curriculum content more clearly from the universities in terms of scope and content.

However, the disperse results underline the statement of Merkle, who already stated in 2006 that despite the Bologna Process, supranational comparability of training programmes is difficult [6]. Another insight of the study is that, however, many participants in the survey are not familiar with the relationship between ECTS credits and hours in teaching. Researchers must take this essential equivalence ratio between hours and ECTS credits into account when planning further surveys.

**Figure 3:**
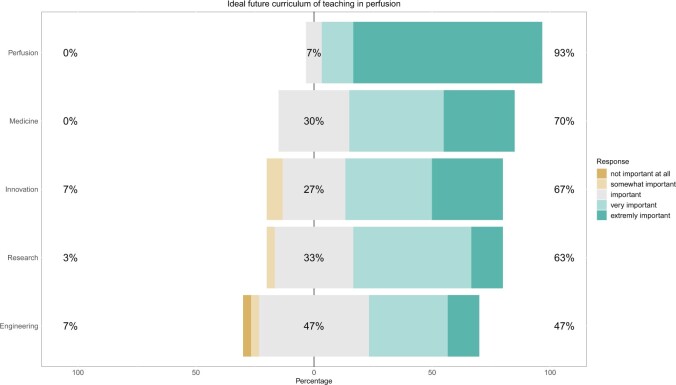
Likert scale for representing core competencies for an ideal curriculum graphically.

The statements on the important key competences are highly interesting (Fig. 3). The trend away from technical, scientific and innovative competences, which is clearly visible in the Likert scale, is contrary to the already described advocacy of academic education. If perfusion schools already attach so little importance to non-medical competencies, the clinics’ point of view would be very interesting. This attitude reveals that people are no longer aware of the fact that, according to Merkle, the invention of the profession of clinical perfusion is closely linked to the technical achievements of the last century [4]. This less interest on scientific and technical skills should be discussed in the profession community even because it further contradicts the statements of Likosky, who claims that science and research should serve as a framework for clinical perfusion [14].

**Figure 4:**
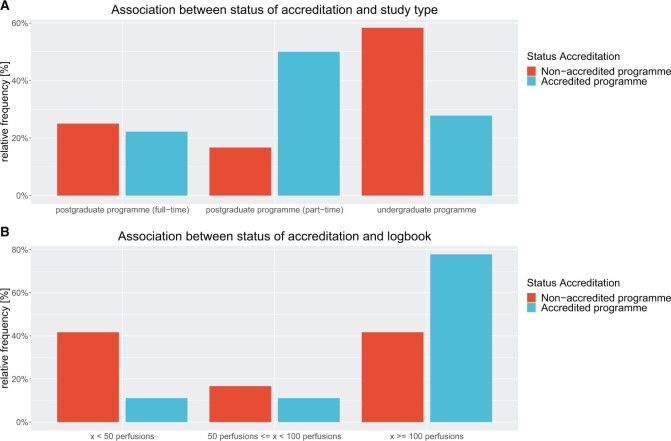
(**A**) Plot of relative frequencies of dependence on accreditation and study form; (**B**) plot of relative frequencies of dependence on accreditation and minimum of clinical perfusions in the logbook.

## CONCLUSION

The results of the survey provide an overview of the current state of teaching and training in clinical perfusion in Europe and serve as a basis for discussion at the level of the relevant European professional societies. The authors recommend to consider a corridor of 50 ECTS credits as a target for future accreditations of European perfusion schools. Also in order to be able to exclude courses that are too short, in the case of unclear or incorrectly balanced curriculum content. In addition, the reintroduction of central final examinations for all perfusion schools is proposed for further considerations. This would correspond to the approach of other countries worldwide and would at least provide comparable evidence of the level of competences achieved by the graduates [15].

In the absence of any European legal provisions on the profession of perfusionists, it is the responsibility of the representatives of the involved national and international perfusion and medical societies to define and demand more precise the content and scope in clinical perfusion education. Because in a finale, only a well-described and recognized profession in clinical perfusion will ensure safe and high-quality treatment of patients in need of Cardio-pulmonary extracorporeal therapy.

### Limitations

In the course of data collection, the determination of the population has been repeatedly questioned, as the information provided by the national delegates cannot be independently verified. A loss of validity must also be assumed because questions were misunderstood or (despite some explanations) not explained in more detail. It must also be assumed that not all participants are sufficiently skilled in the English language and therefore did not understand questions correctly. Some participants gave such contradictory information about for instance ECTS credits in clinical perfusion that it is hardly possible to reconstruct and evaluate the results.

## Data Availability

The survey data are part of a master’s thesis at Furtwangen University in collaboration with the European Board of Cardiovascular Perfusion. The data underlying this article will be shared on reasonable request to the corresponding author.

## ABBREVIATIONS

DGfK: German Society for Cardiovascular Engineering
EACTS: European Association for Cardio Thoracic Surgery
EBCP: European Board of Cardiovascular Perfusion
ECCP: European Certificate in Cardiovascular Perfusion
ECMO: Extra Corporeal Membrane Oxygenation
ECTS: European Credit Transfer System
EQF: European Qualifications Framework
